# Proteolytic Disassembly of Viral Outer Capsid Proteins Is Crucial for Reovirus-Mediated Type-I Interferon Induction in Both Reovirus-Susceptible and Reovirus-Refractory Tumor Cells

**DOI:** 10.1155/2015/468457

**Published:** 2015-03-19

**Authors:** Yuki Katayama, Yuichi Terasawa, Masashi Tachibana, Hiroyuki Mizuguchi, Fuminori Sakurai

**Affiliations:** ^1^Laboratory of Biochemistry and Molecular Biology, Graduate School of Pharmaceutical Sciences, Osaka University, 1-6 Yamadaoka, Suita, Osaka 565-0871, Japan; ^2^Laboratory of Hepatic Differentiation Research, National Institute of Biomedical Innovation, 7-6-8 Asagi, Saito, Ibaraki, Osaka 567-0085, Japan; ^3^Center for Advanced Medical Engineering and Informatics, Osaka University, 1-6 Yamadaoka, Suita, Osaka 565-0871, Japan; ^4^Laboratory of iPS Research, Graduate School of Pharmaceutical Sciences, Osaka University, 1-6 Yamadaoka, Suita, Osaka 565-0871, Japan; ^5^Laboratory of Regulatory Sciences for Oligonucleotide Therapeutics, Clinical Drug Development Unit, Graduate School of Pharmaceutical Sciences, Osaka University, 1-6 Yamadaoka, Suita, Osaka 565-0871, Japan

## Abstract

Oncolytic reovirus induces innate immune responses, which contribute to the antitumor activity of reovirus, following *in vivo* application. Reovirus-induced innate immune responses have been relatively well characterized in immune cells and mouse embryonic fibroblasts cells; however, the mechanisms and profiles of reovirus-induced innate immune responses in human tumor cells have not been well understood. In particular, differences in reovirus-induced innate immune responses between reovirus-susceptible and reovirus-refractory tumor cells remain unknown, although the intracellular trafficking of reovirus differs between these tumor cells. In this study, we examined reovirus-induced upregulation of interferon- (IFN-) *β* and of the proapoptotic gene, Noxa, in reovirus-susceptible and -refractory tumor cells. IFN-*β* and Noxa were significantly induced by reovirus *via* the IFN-*β* promoter stimulator-1 (IPS-1) signaling in both types of tumor cells. Inhibition of cathepsins B and L, which are important for disassembly of reovirus outer capsid proteins and escape into cytoplasm, largely suppressed reovirus-induced upregulation of IFN-*β* and Noxa expression in not only reovirus-susceptible but also reovirus-refractory tumor cells. These results indicated that in both reovirus-susceptible and reovirus-refractory tumor cells, disassembly of the outer capsid proteins by cathepsins and the escape into the cytoplasm were crucial steps for reovirus-induced innate immunity.

## 1. Introduction

Mammalian orthoreovirus (reovirus), which is a nonenveloped virus family possessing a double-stranded RNA (dsRNA) genome, is ubiquitous in the environment and is nonpathogenic to adults [1]. Among mammalian reoviruses, reovirus type 3 Dearing (T3D) specifically replicates in cancer cells, resulting in efficient tumor cell lysis but not in normal tissues. Reovirus has gained much attention as an oncolytic agent and has already progressed into several clinical trials, including phase 3 clinical trials, for different types of tumors [2].

Reovirus infection is initiated by attachment to the receptor, junctional adhesion molecule A (JAM-A), on the cell surface, followed by internalization into cells* via* the endocytic pathway [3–6]. In late endosomes/lysosomes, reovirus virions are disassembled mainly by cathepsins B and L, producing infectious/intermediate subviral particle (ISVP) [7–11]. ISVP penetrates the membrane of the late endosomes/lysosomes into the cytoplasm. Viral transcripts are translated in the cytoplasm, producing progeny virus particles. Among the infection steps described above, disassembly of the virus outer capsid proteins by cathepsins has been demonstrated to be crucial for tumor cell-specific reovirus replication [12, 13]. Disassembly of the outer capsid proteins by cathepsins and subsequent invasion into the cytoplasm are limited in reovirus-refractory tumor cells, which often show low activity levels of cathepsins B and L.

There are several reports of reovirus-induced innate immune responses in mouse embryonic fibroblasts and immune cells, including dendritic cells (DCs) [14, 15]. Following internalization, the reovirus double-stranded RNA (dsRNA) genome and/or viral transcripts are recognized by RNA sensors in the cytoplasm, resulting in the production of inflammatory cytokines and type I interferons (IFNs). Retinoic acid-inducible gene-I (RIG-I), melanoma differentiation-associated gene 5 (MDA5), and RIG-I-like DExD/H helicases, such as DDX1–DDX21–DHX36 complexes, DHX9, and DDX60, are involved in reovirus-mediated innate immunity in mouse embryonic fibroblasts and immune cells [16–19]. On the other hand, reovirus is expected to induce innate immune responses even in tumor cells. In addition, reovirus-mediated innate immunity would be involved, at least in part, in reovirus-mediated tumor cell killing. Knowlton et al. reported that RIG-I-mediated signaling directly induces expression of Noxa, which is a proapoptotic BH3-domain-only protein of the Bcl-2 family,* via* IFN regulatory transcription factor- (IRF-) 3 and NF-*κ*B [20]. Expression of Noxa is upregulated following infection with reovirus in tumor cells; however, the profiles and mechanisms of reovirus-mediated innate immune responses, including Noxa induction, in tumor cells are not fully understood. In particular, it remains to be clarified whether the mechanisms of reovirus-mediated innate immunity differ between reovirus-susceptible and reovirus-refractory tumor cells, although the sensitivities to reovirus differ between tumor cell lines. As described above, disassembly of the outer capsid proteins and subsequent escape into the cytoplasm efficiently proceed in reovirus-susceptible tumor cells, whereas these steps are less efficient in reovirus-refractory tumor cells. This difference made us hypothesize that the mechanisms of reovirus-induced innate immune responses differed between reovirus-susceptible and reovirus-refractory tumor cells. Differences in intracellular trafficking of innate immune ligands contribute to differences in the mechanism and levels of innate immune activation [21, 22]. Elucidation of reovirus-mediated innate immune responses in tumor cells will be crucial to the evaluation of the mechanism of tumor cell-specific cell lysis and of the safety profile of reovirus.

In this study, we examined reovirus-mediated induction of type I IFN and Noxa in reovirus-susceptible and reovirus-refractory tumor cells. We revealed that IFN-*β* and Noxa expressions were induced by reovirus in both reovirus-susceptible and reovirus-refractory tumor cells mainly* via* the RIG-I/IFN-*β* promoter stimulator-1 (IPS-1) pathway. In addition, disassembly of the outer capsid proteins by cathepsins B and L was a crucial step for reovirus-induced IFN-*β* production not only in reovirus-susceptible tumor cells with high activity levels of cathepsins B and/or L, but also in reovirus-refractory tumor cells with low cathepsins B and/or L.

## 2. Materials and Methods

### 2.1. Cell Lines

A549 (a human lung carcinoma cell line), A431 (a human epidermoid carcinoma cell line), and HepG2 (a human hepatocellular carcinoma cell line) cells were cultured with Dulbecco's modified Eagle's medium. H1299 (a human non-small-cell lung cancer cell line) cells were cultured with RPMI 1640 medium. All mediums described above were supplemented with 10% fetal bovine serum (FBS), streptomycin (100 *μ*g/mL), and penicillin (100 U/mL). L929 (a mouse fibroblast cell line) cells were cultured with minimum essential medium supplemented with 5% FBS, streptomycin (100 *μ*g/mL), and penicillin (100 U/mL).

### 2.2. Reovirus

Reovirus T3D was amplified in L929 cells and purified by CsCl density gradient centrifugation as previously described [23]. Viral titers were determined by a plaque assay using L929 cells.

### 2.3. Small Interfering RNA-Mediated Knockdown of RNA Sensors

Cells were plated in a 24-well plate. The following day, cells were transfected with small interfering RNAs (siRNAs) using Lipofectamine RNAiMAX Transfection Reagent (Invitrogen, Carlsbad, CA) according to the manufacturer's instructions. siRNAs for RIG-I, DDX1, DDX3, DDX60, and DHX33 were purchased from Dharmacon (Lafayette, CO) and were transfected at final concentrations of 30 nM. siRNAs for IPS-1, TLR3, TRIF, MyD88, DHX9, and RKR were purchased from Gene Design (Osaka, Japan). siRNA for MDA5 was purchased from Invitrogen, and control siRNA was purchased from Qiagen (Valencia, CA). They were transfected at final concentrations of 50 nM. Forty-eight hours after siRNA transfection, reovirus was added to the cells at multiplicities of infection (MOI) of 20. mRNA levels of IFN-*β* and Noxa were determined by quantitative RT-PCR (qRT-PCR) 24 hrs after infection.

### 2.4. qRT-PCR Analysis

Total RNA was extracted from cells using ISOGEN (Nippon Gene, Tokyo, Japan). After the treatment with RNase-free DNase I (New England Biolabs, Ipswich, MA), complementary DNA (cDNA) was synthesized from 1 *μ*g of total RNA using the Superscript VILO cDNA synthesis kit (Invitrogen). THUNDERBIRD qPCR Mix (TOYOBO, Osaka, Japan) was used for qRT-PCR. The mRNA levels of indicated genes were normalized by glyceraldehyde 3-phosphate dehydrogenase (GAPDH) mRNA levels. Sequences of the primers used for qRT-PCR are available on request.

### 2.5. Preparation of ISVP and Effects of Cathepsin Inhibition on Reovirus-Induced Innate Immunity

ISVP was prepared as previously described [7]. Briefly, reovirus was digested with 200 *μ*g/mL of N-p-tosyl-l-lysine chloromethyl ketone-treated chymotrypsin (CHT) (Sigma Aldrich, St. Louis, MO) in a buffer containing 15 mM sodium citrate and 75 mM NaCl (pH 7.5) at 37°C for 25 minutes. The entire reaction was terminated by the addition of 2 mM phenylmethylsulfonyl fluoride and the incubation of reaction mixtures on ice. Production of ISVP was confirmed by SDS-PAGE and coomassie brilliant blue staining.

For the inhibition of cathepsins B and L, cells plated in a 24-well plate were preincubated for 1 hr in medium containing 10 *μ*M cathepsin B inhibitor CA-074Me (Millipore, Billerica, MA) and/or cathepsin L inhibitor III (Millipore). Cells were then infected with reovirus or ISVP at a concentration equivalent to an MOI of 20. Total RNA was recovered 24 hrs after infection, and subsequently qRT-PCR analysis was performed as described above.

### 2.6. Transfection with Reovirus Genome and 5′-Triphosphate Double-Stranded RNA

The reovirus genome was recovered from the purified virus particles using the RNeasy Mini Kit (Qiagen) according to the manufacturer's instructions. Isolated reovirus genome was transfected to cells pretreated with the cathepsin inhibitors using Lipofectamine 2000 Transfection Reagent (Invitrogen) at 200 ng/mL. A RIG-I agonist 5′-triphosphate double-stranded RNA (5′-ppp dsRNA) was purchased from InvivoGen (San Diego, CA) and similarly transfected at 1 *μ*g/mL. Total RNA was recovered 24 hrs after transfection, and subsequently qRT-PCR analysis was performed as described above.

### 2.7. Preparation of Ultraviolet- (UV-) Inactivated Reovirus (UV-reo) and Effects on Reovirus-Induced Innate Immunity

UV-reo was prepared by exposing live virus to UV light for 20 minutes. Loss of infection ability of reovirus was confirmed by a plaque assay using L929 cells. Cells were infected with reovirus or UV-reo at a concentration equivalent to an MOI of 20. Total RNA was recovered 24 hrs after infection, and subsequently qRT-PCR analysis was performed as described above.

### 2.8. Statistical Analysis

Statistical significance was determined using Student's *t*-test. Data are presented as the means ± SD.

## 3. Results

### 3.1. Reovirus-Mediated Induction of IFN-*β* and Noxa in Reovirus-Susceptible and Reovirus-Refractory Tumor Cells

**Figure 1:**
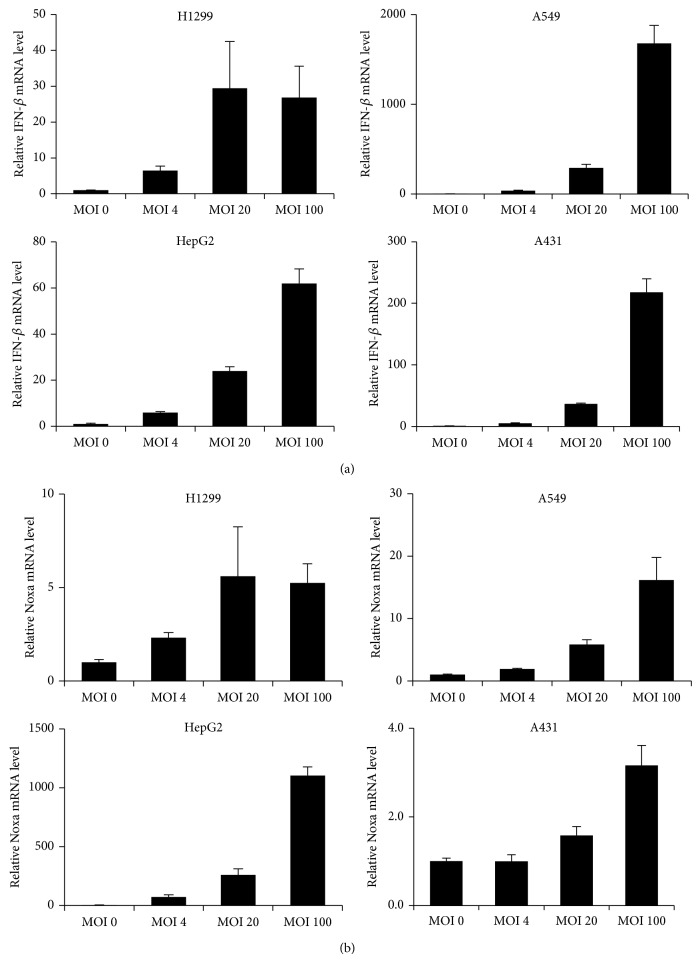
Reovirus-mediated induction of IFN-*β* and Noxa in tumor cells. (a), (b) Dose-dependent induction of IFN-*β* and Noxa expression by reovirus. qRT-PCR analysis was used to measure mRNA levels of IFN-*β* and Noxa 24 hrs after infection. The data were normalized by the data of the mock group. The data shown represent the mean ± SD of triplicate measurements.

Previous studies, including ours, demonstrated that cell viabilities following reovirus infection largely differed between tumor cell lines [8, 11, 24, 25]. In this study, we used H1299 and HepG2 cells as reovirus-susceptible tumor cells and A549 and A431 cells as reovirus-refractory tumor cells, according to our previous study [26]. The activity levels of cathepsin B and/or L, which are cysteine proteases crucial for the disassembly of reovirus outer capsid proteins *σ*3 and *μ*1 and subsequent penetration into the cytoplasm [27, 28], were more than 2-fold higher in H1299 and HepG2 cells than in A549 and A431 cells. We examined reovirus-mediated induction levels of IFN-*β* and Noxa in the reovirus-susceptible and reovirus-refractory tumor cells 24 hrs after infection. The IFN-*β* mRNA levels reached a peak 24 hrs after infection (data not shown). qRT-PCR analysis demonstrated that IFN-*β* mRNA levels were significantly elevated in a dose-dependent manner in all the tumor cells examined, including reovirus-refractory tumor cells, following reovirus infection (Figure 1(a)). The highest IFN-*β* mRNA levels by reovirus were found in A549 cells, followed by A431 cells. mRNA levels of Noxa, whose induction involves the RIG-I/IPS-1 signaling pathway, were most highly elevated in HepG2 cells. The lowest level of Noxa mRNA was found in A431 cells following the addition of reovirus (an approximately 3-fold increase at an MOI of 100). The Noxa mRNA levels were comparable in H1299 and A549 cells (Figure 1(b)). These results indicated that reovirus induced IFN-*β* and Noxa expression in both reovirus-susceptible and reovirus-refractory tumor cells.

### 3.2. Roles of RNA Sensors in Reovirus-Mediated Induction of IFN-*β* and Noxa

**Figure 2:**
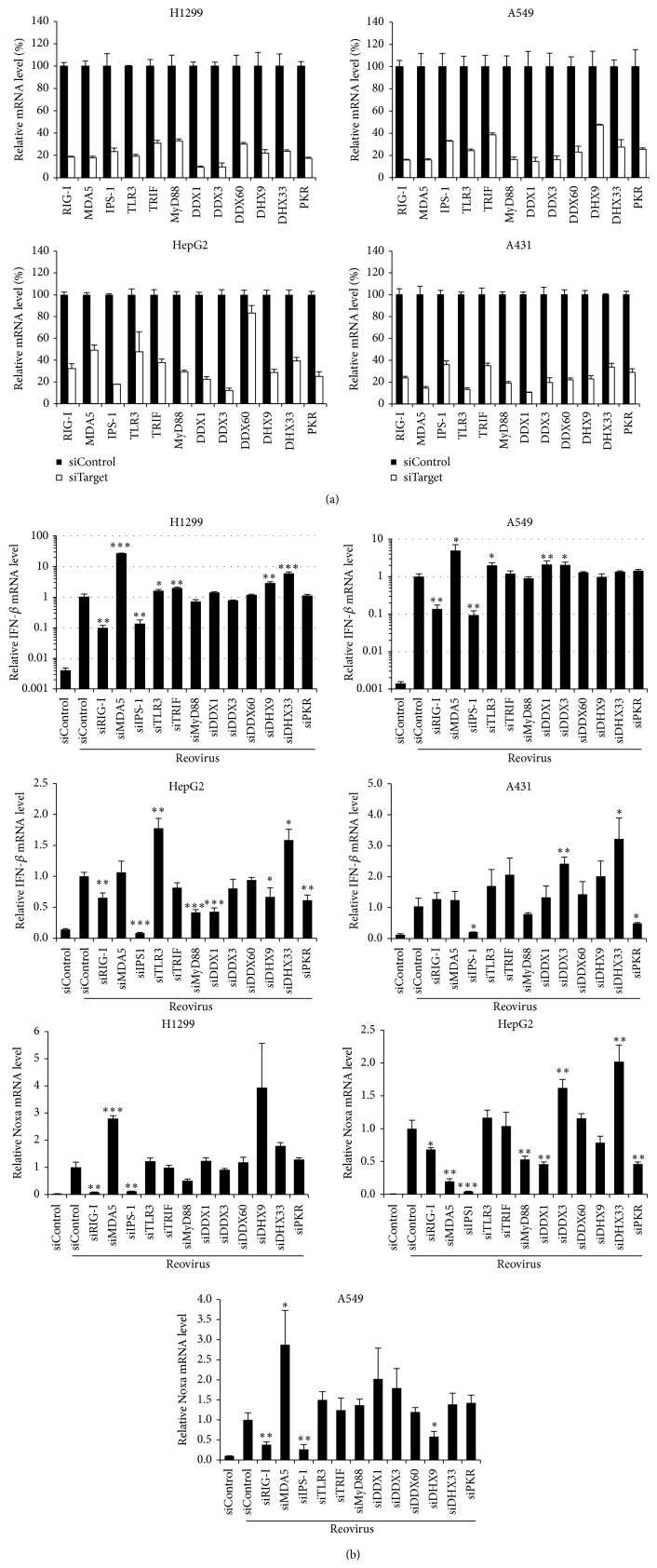
Effects of knockdown of RNA sensors and their adaptor molecules on reovirus-induced expression of IFN-*β* and Noxa in tumor cells. (a) Knockdown efficiencies of siRNAs. Cells were transfected with siRNAs targeting indicated gene or control siRNA (siControl). Following a 48-hour incubation, cells were harvested for the evaluation of siRNA-mediated knockdown efficiencies by qRT-PCR analysis. (b) mRNA levels of IFN-*β* and Noxa following reovirus infection in the cells pretreated with siRNAs. Forty-eight hours after transfection with siRNAs, cells were infected with reovirus at an MOI of 20. Cells were harvested 24 hrs after infection for analysis of IFN-*β* and Noxa mRNA levels by qRT-PCR analysis. The data were normalized by the data of the siControl group infected with reovirus. The data shown represent the mean ± SD of triplicate measurements. ^*^ *P* < 0.05, ^**^ *P* < 0.01, and ^***^ *P* < 0.001, compared with the siControl group infected with reovirus.

Next, in order to examine which RNA sensors are involved in reovirus-mediated induction of IFN-*β* and Noxa in tumor cells, tumor cells were transfected with siRNAs against various types of RNA sensors, followed by infection with reovirus. Although several studies demonstrated that the reovirus genome is recognized by cytoplasmic dsRNA sensors RIG-I and MDA5 in immune cells and mouse embryonic fibroblasts [15], DExD/H box helicase families, which are involved in the recognition of double-stranded RNA, were recently identified [16–19]. Expression of the RNA sensor genes was knocked down significantly, by more than 50%, following siRNA transfection (Figure 2(a)). We confirmed that treatment with siRNA alone did not induce upregulation of IFN-*β* mRNA levels (data not shown). Compared with the control siRNA group, reovirus-induced IFN-*β* mRNA levels were clearly decreased in the cells transfected with siRNAs against RIG-I (siRIG-I) and IPS-1 (siIPS-1) in H1299 and A549 cells (Figure 2(b)). In HepG2 cells, knockdown of not only RIG-I and IPS-1 but also MyD88, DDX1, DHX9, and PKR resulted in a significant decrease of IFN-*β* mRNA levels. In A431 cells, reovirus-induced IFN-*β* mRNA levels were decreased by knockdown of IPS-1 and PKR but not RIG-I. In all the cells examined, reovirus-induced upregulation of IFN-*β* expression was most largely suppressed by knockdown of IPS-1 among the molecules examined in this study. In contrast, IFN-*β* mRNA levels were comparable or higher in the cells transfected with siRNAs against MDA5 and several of the RNA sensors and adaptor molecules compared to those in the cells transfected with control siRNAs. Noxa mRNA profiles were similar to those of IFN-*β* mRNA in H1299, HepG2, and A549 cells, although pretreatment with siRNAs against the DExD/H box helicase families did not result in significant increases in reovirus-induced Noxa mRNA levels in H1299 and A549 cells. Knockdown of IPS-1 induced the largest reduction in the Noxa mRNA levels in all three cell lines. These results indicated that, regardless of susceptibility to reovirus, the IPS-1-dependent pathway was mainly involved in reovirus-mediated induction of IFN-*β* and Noxa in tumor cells. Effects of knockdown of RNA sensors and adaptor molecules on reovirus-mediated Noxa induction were not examined in A431 cells, because infection with reovirus at an MOI of 20 did not result in a statistically significant elevation in the Noxa mRNA levels in A431 cells, as shown in Figure 1.

### 3.3. Role of Cathepsins B and L on Reovirus-Mediated Induction of IFN-*β* and Noxa

**Figure 3:**
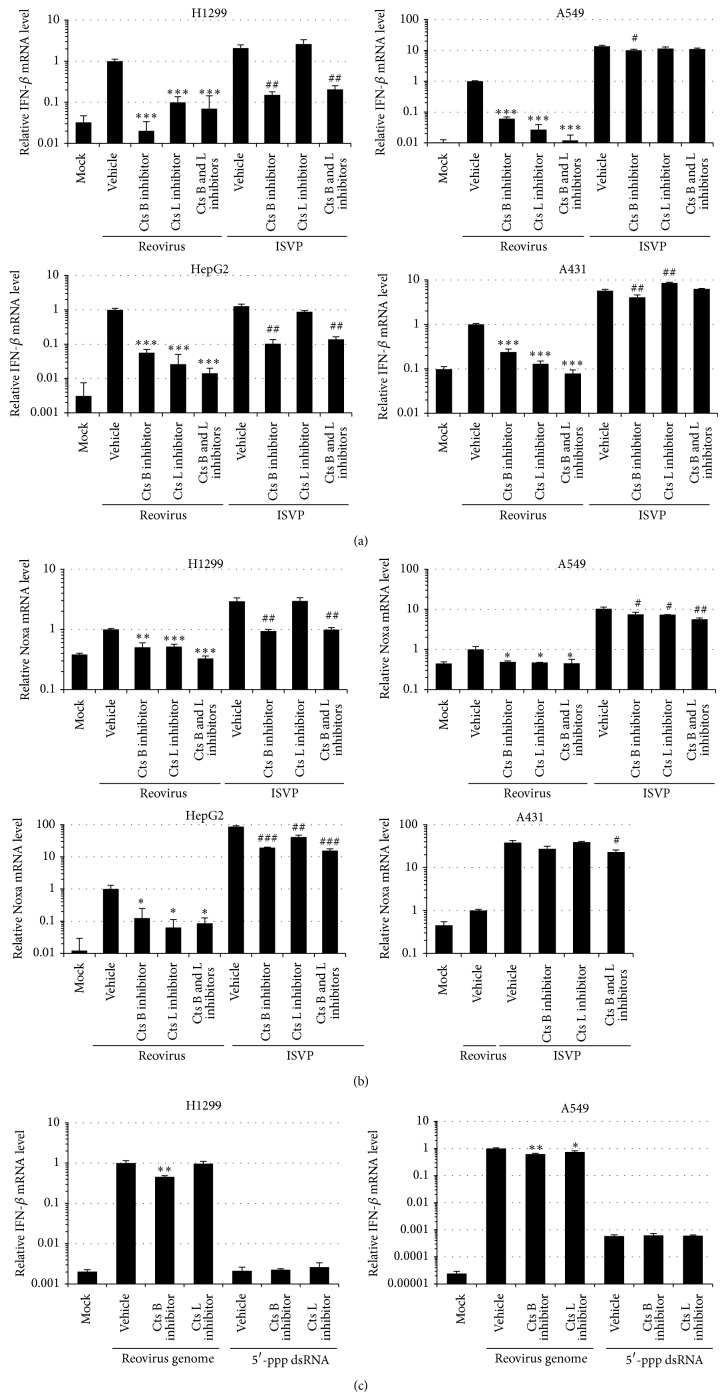
Effects of inhibition of cathepsins B and L on reovirus-mediated induction of IFN-*β* and Noxa in tumor cells. (a), (b) mRNA levels of IFN-*β* and Noxa following reovirus or ISVP infection in the cells pretreated with cathepsin inhibitors. Cells were preincubated for 1 hr in the medium supplemented with 10 *μ*M of inhibitors of cathepsins B and L. Cells were infected with reovirus or ISVP in equivalent doses for an MOI of 20. Data were normalized by the data of the nopretreatment/reovirus group. (c) mRNA levels of IFN-*β* and Noxa following transfection with the reovirus genome (200 ng/mL) and 5′-ppp dsRNA (1 *μ*g/mL). Cells were harvested for the evaluation of IFN-*β* and Noxa mRNA levels by qRT-PCR analysis following a 24-hour incubation. Data were normalized by the data of the nopretreatment/reovirus genome group. The data shown represent the mean ± SD of triplicate measurements. Cts, cathepsin. ^*^ *P* < 0.05, ^**^ *P* < 0.01, compared with the nopretreatment/reovirus or reovirus genome group.

In order to examine whether proteolytic disassembly of the reovirus outer capsid proteins and penetration from late endosomes/lysosomes into the cytoplasm were required for the induction of type I IFN in not only reovirus-susceptible but also reovirus-refractory tumor cells, reovirus or infectious subviral particles (ISVP), which are produced by the proteolysis of the outer capsid proteins and can penetrate the membranes of late endosomes/lysosomes into the cytoplasm [29–31], were added to the cells after incubation with inhibitors of cathepsin B and/or L. The induction levels of IFN-*β* mRNA in ISVP-treated cells were comparable or higher than those in reovirus-infected cells in both reovirus-susceptible cells and reovirus-refractory cells (Figures 3(a) and 3(b)). The differences in the mRNA levels of IFN-*β* following treatment with reovirus and ISVP were larger in A549 and A431 cells than in H1299 and HepG2 cells. Following reovirus infection, IFN-*β* mRNA levels significantly decreased, by more than 75% in the presence of either cathepsin B inhibitor or cathepsin L inhibitor in the tumor cells, regardless of susceptibility to reovirus. Cathepsin inhibitors induced a larger reduction in the IFN-*β* mRNA levels upon addition of reovirus than upon addition of ISVP, although cathepsin B inhibitor also reduced the ISVP-mediated upregulation in the IFN-*β* mRNA levels. Pretreatment with the cathepsin L inhibitor did not reduce ISVP-mediated IFN-*β* induction. The induction levels of Noxa mRNA in ISVP-treated cells were also higher than those in reovirus-infected cells in both reovirus-susceptible cells and reovirus-refractory cells (Figure 3(b)). Reovirus- or ISVP-induced Noxa mRNA levels also declined by pretreatment with the cathepsin inhibitors in both types of cell lines, although the cathepsin inhibitor-mediated reduction in the Noxa mRNA levels was lower than that in the IFN-*β* mRNA levels. These results indicated that disassembly of the outer capsid proteins by cathepsins B and L followed by membrane penetration were important for reovirus-induced IFN-*β* and Noxa expression in both reovirus-susceptible and reovirus-refractory cells.

In order to examine whether cathepsin inhibitors affect the IFN-*β* induction by the RIG-I/IPS-1 signaling pathway, reovirus genome and 5′-ppp dsRNA, which is a RIG-I ligand, were transfected in the cells in the presence of cathepsin inhibitors. IFN-*β* induction was mediated following transfection with the purified reovirus genome in the cells (Figure 3(c)). Treatment with cathepsin B inhibitor slightly, but significantly, suppressed IFN-*β* induction following transfection with reovirus genome in both H1299 and A549 cells. Inhibition of cathepsin L also slightly reduced IFN-*β* mRNA levels by the reovirus genome in A549 cells; however, the effects of the cathepsin inhibitors on reovirus genome-induced IFN-*β* expression were much less than those on reovirus-induced IFN-*β* expression. 5′-ppp dsRNA also mediated IFN-*β* induction in A549 cells, although no significant elevations in IFN-*β* mRNA levels were observed in H1299 cells. Neither of the cathepsin inhibitors reduced the 5′-ppp dsRNA-mediated induction of IFN-*β* expression in A549 cells. These results suggested that the cathepsin inhibitors did not largely inhibit the RIG-I/IPS-1 signaling pathway, although the reovirus genome-induced elevation in IFN-*β* mRNA levels was slightly reduced by the cathepsin inhibitors.

### 3.4. UV-reo Induced IFN-*β* and Noxa Expression

**Figure 4:**
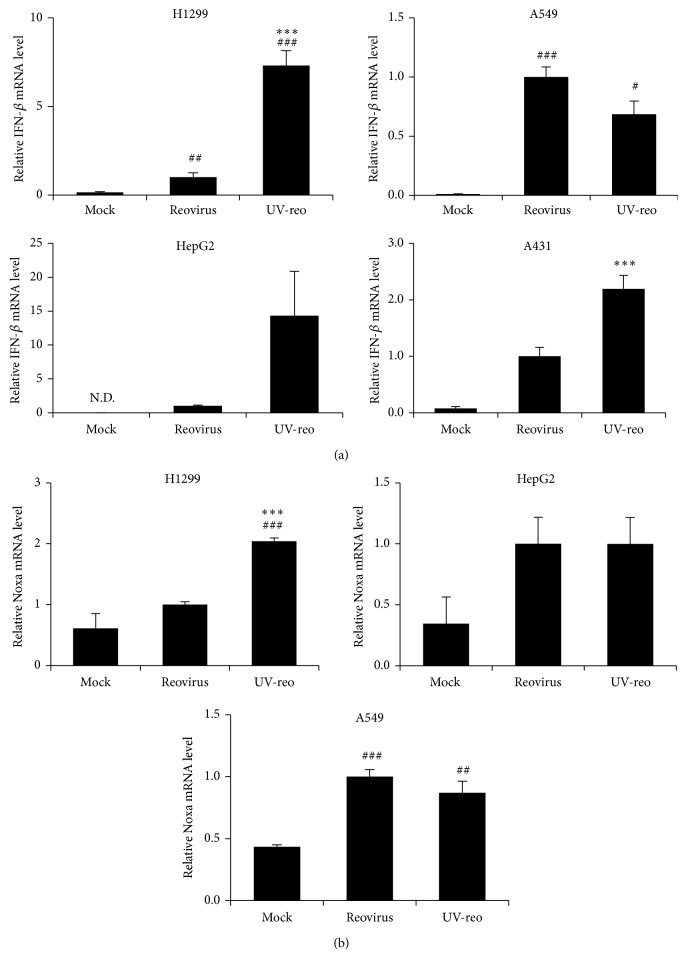
Expression levels of IFN-*β* and Noxa following addition of UV-irradiated reovirus in tumor cells. (a), (b) IFN-*β* and Noxa mRNA levels following the addition of reovirus or UV-reo. Reovirus and UV-reo were added to the cells in equivalent doses for an MOI of 20. Following a 24-hour incubation, cells were harvested for the evaluation of IFN-*β* and Noxa mRNA levels by qRT-PCR analysis. Data were normalized by the data of reovirus group. The data shown represent the mean ± SD of triplicate measurements. ^#^ *P* < 0.05, ^##^ *P* < 0.01, and ^###^ *P* < 0.001, compared with mock group; ^***^ *P* < 0.001, compared with reovirus group.

Furthermore, in order to examine whether replication of virus genomic dsRNA is important for reovirus-mediated induction of IFN-*β* and Noxa, UV-inactivated reovirus (UV-reo) was added to the tumor cells. UV-reo induced slightly lower levels of IFN-*β* than live reovirus, but significantly higher levels of IFN-*β* than in the mock group in A549 cells (Figure 4). By contrast, in HepG2 and H1299 cells, significantly higher levels of IFN-*β* mRNA were found by UV-reo compared with the live reovirus, despite a replication ability defect in the former. UV-reo also induced significantly higher levels of Noxa mRNA, compared with mock-treated cells, in these cell lines. These results indicated that replication of reovirus genome was not necessarily essential for the reovirus-mediated induction of IFN-*β* and Noxa in tumor cells.

## 4. Discussion

The aim of this study is to reveal mechanisms of reovirus-induced innate immune responses in reovirus-susceptible and reovirus-refractory tumor cells. Several reports noted that reovirus induces innate immune responses* via* RIG-I and MDA5 in immune cells, including dendritic cells [14], as well as in mouse embryonic fibroblasts [15]; however, the profiles and mechanisms of reovirus-induced innate immunity in tumor cells have been less characterized. In particular, it remains to be clarified whether the mechanisms of reovirus-mediated innate immunity differ between reovirus-susceptible and reovirus-refractory tumor cells, although the sensitivities to reovirus and intracellular trafficking of reovirus differ between tumor cell lines. Clarification of reovirus-induced innate immunity in tumor cells is crucial for the evaluation of the* in vivo* safety profiles of reovirus and reovirus-mediated oncolysis.

The sensitivity of tumor cell lines against reovirus largely differs between tumor cell lines [8, 11, 24, 25]. Several factors explaining the differences in sensitivity against reovirus, including the activation status of Ras [32], have been reported. Among these factors, activity levels of cathepsins B and L, which are mainly localized in late endosomes/lysosomes and are involved in the disassembly of the outer capsid proteins and the subsequent escape into the cytoplasm, have been demonstrated to be largely responsible for differences in sensitivities to reovirus [8, 12, 13]. Activity levels of cathepsins B and L in reovirus-refractory tumor cells were lower than those in reovirus-susceptible tumor cells, which let us to hypothesize before starting the experiments that the induction levels and mechanisms of reovirus-induced innate immunity differed between reovirus-susceptible and reovirus-refractory tumor cells. For example, we hypothesized that reovirus escaped into the cytoplasm and activated innate immunity* via* RIG-I and/or MDA5 in reovirus-susceptible tumor cells with high cathepsin activity levels. On the other hand, reovirus could not escape from the late endosomes/lysosomes, leading to a limited cytokine response, in reovirus-refractory tumor cells with low cathepsin activity levels. Alternatively, reovirus activated innate immunity* via* TLR families which are located on the endosomal membrane, in reovirus-refractory tumor cells. Adenovirus serotype 2 mutant* ts*1, which is defective in endosomal escape, induced only a limited cytokine response [33]. However, our results indicated that disassembly of the outer capsid proteins by the cathepsins and the subsequent penetration into the cytoplasm occurred even in reovirus-refractory tumor cells, resulting in induction of type-I IFN and Noxa expression mainly* via* RIG-I/IPS-1 signaling, although the amounts of virus genomes penetrating the cytoplasm in reovirus-refractory tumor cells would be lower than those in reovirus-susceptible tumor cells. RIG-I and MDA5, which are localized in the cytoplasm, were involved in reovirus-mediated innate immune responses in immune cells and mouse embryonic fibroblasts [15, 17], indicating that cathepsins B and L are also crucial for reovirus-induced innate immunity in these normal cells.

Knockdown of neither TRIF nor MyD88, both of which are adaptor proteins involved in TLR family-mediated innate immune responses, apparently affected reovirus-induced IFN-*β* production in any of the tumor cell lines examined except HepG2 cells. This indicated that TLR were not involved in reovirus-induced innate immune responses in tumor cells. TLR families are mainly expressed on the cell surface and endosomal membrane [34]. The reovirus genome inside the viral particle has no chance of accessing TLR families on the cellular membrane. In HepG2 cells, reovirus-mediated IFN-*β* induction was significantly reduced by knockdown of TRIF or MyD88, probably because HepG2 cells were highly susceptible to reovirus, and not only dsRNA but also single-stranded RNA (ssRNA) was efficiently produced during the viral genome replication process in HepG2 cells, leading to recognition by the TLR family members.

siRNA-mediated knockdown experiments indicated that the DExD/H box helicase family, including DHX9 and DDX1–DDX21–DHX36 complexes, was not involved in reovirus-mediated IFN-*β* induction in the tumor cells other than HepG2 cells. Previous studies demonstrated that the DExD/H box helicases were involved in reovirus-induced IFN-*β* production in mouse bone marrow-derived dendritic cells [16–18]. The discrepancies between this study and the previous studies might be due to the differences in the expression levels of DExD/H box helicases in tumor cells and immune cells. In addition, MDA5 did not play an important role in reovirus-mediated IFN-*β* production in the tumor cells. Holm et al. reported that MDA5 did not mediate reovirus-mediated IFN-*β* production through IRF-3/7 activation in 293T cells [35]. On the other hand, Loo et al. demonstrated that both RIG-I and MDA5 were involved in the recognition of the reovirus dsRNA genome and that MDA5 recognized long segments of reovirus genome in mouse embryonic fibroblasts [15]. These reports suggest that the mechanism of reovirus-induced innate immunity might differ between cell types. Rather, siRNA-mediated knockdown of MDA5 slightly but significantly enhanced IFN-*β* mRNA levels following the addition of reovirus in this study, probably because higher copy numbers of reovirus genome were recognized by RIG-I due to the knockdown of MDA5. In A431 cells, knockdown of IPS-1 significantly reduced the reovirus-induced IFN-*β* expression, but the reovirus-mediated IFN-*β* induction was not reduced by either knockdown of RIG-I or MDA5. RIG-I and MDA5 would be expected to compensate for each other in A431 cells.

The differences in mRNA levels of IFN-*β* between the cells treated with reovirus and those treated with ISVP were larger in the reovirus-refractory cells than in the reovirus-susceptible cells. ISVP penetrates into the cytoplasm without cathepsin-mediated disassembly of the outer capsid in the endosomes/lysosomes even in reovirus-refractory cells, although reovirus-refractory cells do not allow efficient penetration of reovirus particles into the cytosol following infection due to the low level of cathepsin activities. In reovirus-susceptible cells, however, both reovirus and ISVP efficiently invade the cytosol due to the high level of cathepsin activities, leading to efficient induction of innate immunity. On the other hand, the difference in the Noxa mRNA levels following treatment with reovirus and ISVP was larger in HepG2 cells than in the reovirus-refractory A549 and A431 cells. Noxa expression is induced by other signals, including p53 [36]. Noxa expression was efficiently induced in HepG2 cells following reovirus infection* via* not only the RIG-I/IPS-1 signaling pathway but also another pathway.

The cathepsin L inhibitor did not reduce the induction of IFN-*β* mRNA following ISVP treatment. On the other hand, the cathepsin B inhibitor suppressed the elevation of IFN-*β* mRNA levels following treatment with ISVP and the reovirus genome, although cathepsin B activity would not be required for escape of the reovirus genome into the cytosol from endosomes in the case that the reovirus genome was transfected using a transfection reagent. Rintahaka et al. reported that dsRNA-stimulated inflammasome activation was abolished by cathepsin inhibitors [37]. Rupture of the release of late endosomes and cathepsin B into the cytosol following adenovirus infection has been shown to activate the NALP3 inflammasome [38]. The reovirus genome might activate inflammasome, and reovirus genome-mediated inflammasome activation and subsequent upregulation of innate immunity might also be suppressed by the cathepsin B inhibitor. Furthermore, it reported that TLR3 is involved in reovirus-induced innate immune responses [39]. Cleavage of TLR3 by cathepsin B is essential for the signaling [40]. Reovirus-induced innate immune responses* via* TLR3 might be inhibited by the cathepsin B inhibitor, although siRNA-mediated knockdown of TLR3 did not apparently decrease mRNA levels of IFN-*β* and Noxa following treatment with reovirus in tumor cells.

In summary, this study demonstrated that disassembly of the outer capsid proteins and late endosome/lysosome escape was crucial for reovirus-mediated induction of IFN-*β* and Noxa in not only reovirus-susceptible tumor cells but also reovirus-refractory tumor cells. In addition, we demonstrated that the RIG-I/IPS-1 signaling pathway, not MDA5, was mainly involved in reovirus-mediated innate immune responses in tumor cells. This study provides important clues for the elucidation of reovirus-mediated oncolysis.
